# 3,6-Di-4-pyridyl-1,4-dihydro-1,2,4,5-tetra­zine

**DOI:** 10.1107/S160053680801742X

**Published:** 2008-06-13

**Authors:** Hai Wang, Hua-Ze Dong, Ning Lu, Hai-Bin Zhu

**Affiliations:** a Department of Chemistry and Chemical Engineering, Southeast University, Nanjing, People’s Republic of China; b Department of Chemistry and Chemical Engineering, State Key Laboratory of Coordination Chemistry, Nanjing University, Nanjing, People’s Republic of China

## Abstract

The mol­ecule of the title compound, C_12_H_10_N_6_, which is V-shaped due to the boat conformation of the dihydro­tetra­zine ring, has crystallographic *C*_2_ symmetry. The dihedral angle between the planes of the two pyridine rings is 31.57 (3)°. Mol­ecules are linked by weak N—H⋯N and C—H⋯N hydrogen bonds, forming a two-dimensional polymeric structure.

## Related literature

For related structures, see: Bradford *et al.* (2004 ▶); Caira *et al.* (1976 ▶); Liou *et al.* (1996 ▶); Zachara *et al.* (2004 ▶); Rao & Hu (2005 ▶). For related literature on tetra­zines, see: Sauer (1996 ▶).
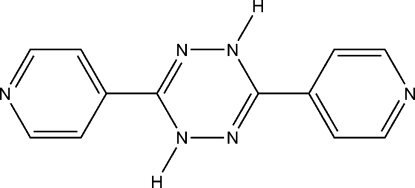


## Experimental

### 

#### Crystal data

C_12_H_10_N_6_
*M* *_r_* = 238.26
Orthorhombic, 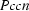

*a* = 11.2862 (18) Å
*b* = 14.481 (2) Å
*c* = 6.8864 (12) Å
*V* = 1125.4 (3) Å^3^
*Z* = 4
Mo *K*α radiation
μ = 0.09 mm^−1^
*T* = 293 (2) K
0.50 × 0.10 × 0.10 mm

#### Data collection

Bruker SMART CCD area-detector diffractometer
Absorption correction: multi-scan (*SADABS*; Bruker, 2000 ▶) *T*_min_ = 0.955, *T*_max_ = 0.991
4214 measured reflections
1105 independent reflections
938 reflections with *I* > 2σ(*I*)
*R*_int_ = 0.032

#### Refinement

*R*[*F* ^2^ > 2σ(*F* ^2^)] = 0.050
*wR*(*F* ^2^) = 0.128
*S* = 1.08
1105 reflections
86 parameters
H atoms treated by a mixture of independent and constrained refinement
Δρ_max_ = 0.20 e Å^−3^
Δρ_min_ = −0.14 e Å^−3^

### 

Data collection: *SMART* (Bruker, 2000 ▶); cell refinement: *SMART*; data reduction: *SAINT* (Bruker, 2000 ▶); program(s) used to solve structure: *SHELXS97* (Sheldrick, 2008 ▶); program(s) used to refine structure: *SHELXL97* (Sheldrick, 2008 ▶); molecular graphics: *SHELXTL* (Sheldrick, 2008 ▶); software used to prepare material for publication: *SHELXTL*.

## Supplementary Material

Crystal structure: contains datablocks I, global. DOI: 10.1107/S160053680801742X/gk2149sup1.cif

Structure factors: contains datablocks I. DOI: 10.1107/S160053680801742X/gk2149Isup2.hkl

Additional supplementary materials:  crystallographic information; 3D view; checkCIF report

## Figures and Tables

**Table 1:** Hydrogen-bond geometry (Å, °)

*D*—H⋯*A*	*D*—H	H⋯*A*	*D*⋯*A*	*D*—H⋯*A*
N3—H3*B*⋯N1^i^	0.83 (2)	2.35 (2)	3.142 (2)	159.8 (18)
C3—H3*A*⋯N2^ii^	0.93	2.55	3.312 (2)	139
C4—H4*A*⋯N1^iii^	0.93	2.55	3.475 (3)	171

Symmetry codes: (i) 
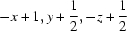
; (ii) 
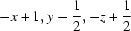
; (iii) 
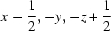
.

**Fig. 1.**
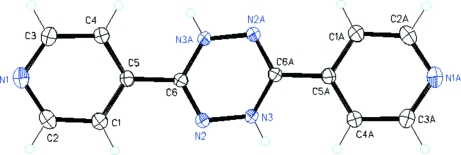
The molecular structure of the title compound with 30% displacement ellipsoids. Symmetry code for the atoms designated with A: -1/2 - x, 1/2 - y, z.

**Fig. 2.**
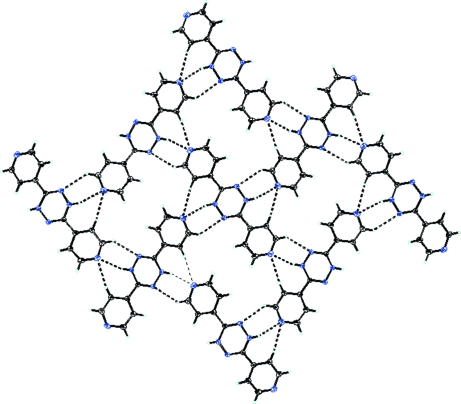
A two-dimensional (4,4) hydrogen-bond network of the title compound viewed along the c axis

## supplementary crystallographic information

### Comment

Tetrazine derivatives have been widely used in pesticides and herbicides as they have a high potential for biological activity and possess a wide range of antiviral and antitumor properties (Sauer, 1996). Herein, we report the crystal structure of a new tetrazine derivative, 3,6-di(pyridin-4-yl)-1,4-dihydro-1,2,4,5-tetrazine.

The molecule of the title compound, which has a crystallographic C_2_ symmetry is shown in Fig. 1. The title compound can be regarded as a V-shaped tetrazine with the dihedral angle between the pyridine rings of 31.57 (3) °. In the crystalline state, each molecule is connected to four adjacent molecules to form a two-dimensional (4,4) hydrogen-bonding network by the intermolecular N—H···N and weak C—H···N hydrogen bonds (Fig. 2.). Crystal structures of several other tetrazine derivatives with a similar shape have been reported (Bradford *et al., *2004; Caira *et al., *1976; Liou *et al.*, 1996; Zachara *et al.*, 2004; Rao & Hu, 2005).

### Experimental

A mixture of 4-cyanopyridine (0.416g, 4.0 mmol), 80% hydrazine hydrate (5 ml), CoCl_2_.6H_2_O (0.238g, 1.0 mmol) and 95% ethanol (4 ml) was heated in a 15-mL Teflon-lined autoclave at 120°C deg for 3 days, followed by slow cooling (5°/h deg) to room temperature. The resulting mixture was washed with 95% ethanol, and red block crystals were collected and dried in air [yield 3.0% (14.3 mg) based on 4-cyanopyridine].

### Refinement

H atoms bonded to N atoms were located in an electron-density difference map and refined isotropically without any restraints. Other H atoms were positioned geometrically and refined using a riding model with C—H = 0.93 Å and with *U*_iso_(H) = 1.2*U*_eq_(C).

### Crystal data

**Table:**

C_12_H_10_N_6_	*F*_000_ = 496
*M**_r_* = 238.26	*D*_x_ = 1.406 Mg m^−^^3^
Orthorhombic, *P**c**c**n*	Mo *K*α radiation λ = 0.71073 Å
Hall symbol: -P 2ab 2ac	Cell parameters from 820 reflections
*a* = 11.2862 (18) Å	θ = 2.5–28.0º
*b* = 14.481 (2) Å	µ = 0.09 mm^−^^1^
*c* = 6.8864 (12) Å	*T* = 293 (2) K
*V* = 1125.4 (3) Å^3^	Block, red
*Z* = 4	0.50 × 0.10 × 0.10 mm

### Data collection

**Table:**

Bruker SMART CCD area-detector diffractometer	1105 independent reflections
Radiation source: fine-focus sealed tube	938 reflections with *I* > 2σ(*I*)
Monochromator: graphite	*R*_int_ = 0.032
Detector resolution: 0 pixels mm^-1^	θ_max_ = 26.0º
*T* = 293(2) K	θ_min_ = 2.8º
φ and ω scans	*h* = −13→10
Absorption correction: multi-scan(SADABS; Bruker, 2000)	*k* = −17→17
*T*_min_ = 0.955, *T*_max_ = 0.991	*l* = −3→8
4214 measured reflections	

### Refinement

**Table:**

Refinement on *F*^2^	Secondary atom site location: difference Fourier map
Least-squares matrix: full	Hydrogen site location: inferred from neighbouring sites
*R*[*F*^2^ > 2σ(*F*^2^)] = 0.050	H atoms treated by a mixture of independent and constrained refinement
*wR*(*F*^2^) = 0.128	*w* = 1/[σ^2^(*F*_o_^2^) + (0.0618*P*)^2^ + 0.3052*P*] where *P* = (*F*_o_^2^ + 2*F*_c_^2^)/3
*S* = 1.08	(Δ/σ)_max_ < 0.001
1105 reflections	Δρ_max_ = 0.20 e Å^−^^3^
86 parameters	Δρ_min_ = −0.14 e Å^−^^3^
Primary atom site location: structure-invariant direct methods	Extinction correction: none

### Special details

**Table:**

Geometry. All e.s.d.'s (except the e.s.d. in the dihedral angle between two l.s. planes) are estimated using the full covariance matrix. The cell e.s.d.'s are taken into account individually in the estimation of e.s.d.'s in distances, angles and torsion angles; correlations between e.s.d.'s in cell parameters are only used when they are defined by crystal symmetry. An approximate (isotropic) treatment of cell e.s.d.'s is used for estimating e.s.d.'s involving l.s. planes.
Refinement. Refinement of *F*^2^ against ALL reflections. The weighted *R*-factor *wR* and goodness of fit *S* are based on *F*^2^, conventional *R*-factors *R* are based on *F*, with *F* set to zero for negative *F*^2^. The threshold expression of *F*^2^ > σ(*F*^2^) is used only for calculating *R*-factors(gt) *etc*. and is not relevant to the choice of reflections for refinement. *R*-factors based on *F*^2^ are statistically about twice as large as those based on *F*, and *R*- factors based on ALL data will be even larger.

### Fractional atomic coordinates and isotropic or equivalent isotropic displacement parameters (Å^2^)

**Table:**

	*x*	*y*	*z*	*U*_iso_*/*U*_eq_	
C1	0.56160 (17)	0.17370 (13)	0.1811 (4)	0.0589 (7)	
H1A	0.5769	0.2351	0.1500	0.071*	
C2	0.65246 (19)	0.11530 (16)	0.2327 (4)	0.0683 (8)	
H2A	0.7286	0.1397	0.2370	0.082*	
C3	0.52968 (17)	−0.00510 (13)	0.2686 (3)	0.0463 (5)	
H3A	0.5172	−0.0671	0.2971	0.056*	
C4	0.43215 (16)	0.04780 (12)	0.2202 (3)	0.0390 (5)	
H4A	0.3571	0.0214	0.2176	0.047*	
C5	0.44678 (15)	0.13961 (11)	0.1762 (3)	0.0322 (4)	
C6	0.34693 (13)	0.20078 (11)	0.1238 (2)	0.0296 (4)	
N1	0.63928 (15)	0.02634 (11)	0.2770 (3)	0.0526 (5)	
N2	0.36287 (11)	0.28776 (9)	0.1283 (2)	0.0331 (4)	
N3	0.26087 (12)	0.33789 (10)	0.0671 (2)	0.0332 (4)	
H3B	0.2708 (17)	0.3931 (14)	0.097 (3)	0.047 (6)*	

### Atomic displacement parameters (Å^2^)

**Table:**

	*U*^11^	*U*^22^	*U*^33^	*U*^12^	*U*^13^	*U*^23^
C1	0.0310 (11)	0.0379 (11)	0.108 (2)	−0.0006 (8)	−0.0039 (11)	0.0119 (11)
C2	0.0282 (11)	0.0536 (13)	0.123 (2)	0.0004 (9)	−0.0073 (12)	0.0094 (14)
C3	0.0396 (13)	0.0355 (10)	0.0639 (14)	0.0077 (8)	−0.0012 (9)	0.0042 (9)
C4	0.0294 (10)	0.0324 (9)	0.0553 (12)	0.0011 (7)	−0.0008 (8)	0.0023 (8)
C5	0.0273 (9)	0.0314 (9)	0.0379 (9)	0.0025 (7)	0.0029 (7)	−0.0026 (7)
C6	0.0255 (9)	0.0272 (8)	0.0360 (9)	−0.0013 (6)	0.0027 (7)	−0.0009 (7)
N1	0.0357 (10)	0.0463 (10)	0.0756 (13)	0.0111 (7)	−0.0021 (8)	0.0028 (9)
N2	0.0238 (8)	0.0283 (7)	0.0471 (9)	0.0007 (6)	0.0036 (6)	0.0006 (6)
N3	0.0270 (8)	0.0239 (7)	0.0487 (9)	0.0011 (6)	0.0019 (6)	0.0030 (6)

### Geometric parameters (Å, °)

**Table:**

C1—C2	1.376 (3)	C4—C5	1.374 (2)
C1—C5	1.387 (2)	C4—H4A	0.9300
C1—H1A	0.9300	C5—C6	1.478 (2)
C2—N1	1.332 (3)	C6—N2	1.273 (2)
C2—H2A	0.9300	C6—N3^i^	1.395 (2)
C3—N1	1.319 (2)	N2—N3	1.4249 (18)
C3—C4	1.382 (3)	N3—C6^i^	1.395 (2)
C3—H3A	0.9300	N3—H3B	0.83 (2)
			
C2—C1—C5	118.94 (18)	C4—C5—C1	116.82 (16)
C2—C1—H1A	120.5	C4—C5—C6	122.84 (15)
C5—C1—H1A	120.5	C1—C5—C6	120.33 (16)
N1—C2—C1	124.8 (2)	N2—C6—N3^i^	121.83 (14)
N1—C2—H2A	117.6	N2—C6—C5	118.64 (15)
C1—C2—H2A	117.6	N3^i^—C6—C5	119.51 (14)
N1—C3—C4	124.48 (18)	C3—N1—C2	115.36 (17)
N1—C3—H3A	117.8	C6—N2—N3	112.51 (13)
C4—C3—H3A	117.8	C6^i^—N3—N2	114.66 (12)
C5—C4—C3	119.61 (17)	C6^i^—N3—H3B	115.7 (14)
C5—C4—H4A	120.2	N2—N3—H3B	107.9 (14)
C3—C4—H4A	120.2		

Symmetry codes: (i) −*x*+1/2, −*y*+1/2, *z*.

### Hydrogen-bond geometry (Å, °)

**Table:**

*D*—H···*A*	*D*—H	H···*A*	*D*···*A*	*D*—H···*A*
N3—H3B···N1^ii^	0.83 (2)	2.35 (2)	3.142 (2)	159.8 (18)
C3—H3A···N2^iii^	0.93	2.55	3.312 (2)	139
C4—H4A···N1^iv^	0.93	2.55	3.475 (3)	171

Symmetry codes: (ii) −*x*+1, *y*+1/2, −*z*+1/2; (iii) −*x*+1, *y*−1/2, −*z*+1/2; (iv) *x*−1/2, −*y*, −*z*+1/2.
